# Placental hormones and the control of fetal growth

**DOI:** 10.1186/1687-9856-2015-S1-O13

**Published:** 2015-04-28

**Authors:** Michael Freemark

**Affiliations:** 1 Duke University Medical Center, Durham, USA

## 

Linear growth in the postnatal period is controlled by the production, secretion, and action of pituitary growth hormone and IGF-1. IGF-I and IGF-II production and signaling are also essential for fetal growth and neural development. But the control of IGF production in the fetus is largely independent of GH.

Among the factors that control fetal IGF production, the availability and utilization of nutrients is most important. Since fetal nutrient supply ultimately derives from maternal nutrient stores, we can understand fetal growth only through analysis of the factors that control maternal nutrient utilization and transport to the fetus.

I will argue that hormones produced by the placenta, including placental lactogen, placental growth hormone, and sex steroids regulate the intake, absorption, utilization and transfer of maternal nutrients to the fetus and modulate fetal and neonatal insulin production. Dysregulation of placental hormone production may be associated with fetal growth retardation or fetal overgrowth and may predispose in later life to glucose intolerance and type 2 diabetes.

